# Assurance of Medical Device Quality with Quality Management System: An Analysis of Good Manufacturing Practice Implementation in Taiwan

**DOI:** 10.1155/2015/670420

**Published:** 2015-05-17

**Authors:** Tzu-Wei Li, Pei-Weng Tu, Li-Ling Liu, Shiow-Ing Wu

**Affiliations:** ^1^Office of Medical Device Evaluation, Center for Measurement Standards, Industrial Technology Research Institute, 321 Sec. 2, Kuang Fu Road, Hsinchu 30011, Taiwan; ^2^Division of Medical Devices and Cosmetics, Food and Drug Administration, 161-2 Kunyang Street, Nangang District, Taipei City 11561, Taiwan; ^3^Division of Medicinal Products, Food and Drug Administration, 161-2 Kunyang Street, Nangang District, Taipei City 11561, Taiwan; ^4^Food and Drug Administration, 161-2 Kunyang Street, Nangang District, Taipei City 11561, Taiwan

## Abstract

The implementation of an effective quality management system has always been considered a principal method for a manufacturer to maintain and improve its product and service quality. Globally many regulatory authorities incorporate quality management system as one of the mandatory requirements for the regulatory control of high-risk medical devices. The present study aims to analyze the GMP enforcement experience in Taiwan between 1998 and 2013. It describes the regulatory implementation of medical device GMP requirement and initiatives taken to assist small and medium-sized enterprises in compliance with the regulatory requirement. Based on statistical data collected by the competent authority and industry research institutes, the present paper reports the growth of Taiwan local medical device industry after the enforcement of GMP regulation. Transition in the production, technologies, and number of employees of Taiwan medical device industry between 1998 and 2013 provides the competent authorities around the world with an empirical foundation for further policy development.

## 1. Introduction

An effective quality management system has always been considered to be a good strategy for a manufacturer to maintain and improve its product and service quality. The Food and Drug Administration of the United States of America (the US FDA) was the first competent authority for medical device to mandate medical device quality system requirement to ensure the safety and effectiveness of medical devices. The US FDA issued a final rule in the Federal Register of July 21, 1978 (43 FR 31 508), prescribing CGMP (current good manufacturing practice) requirements for medical devices. This quality system requirement provided the government and industry with a foundation for ensuring that medical devices manufactured comply with the established specifications and with continuous improvement strategy. The quality system regulation also requires establishment of documentation and records for investigating quality problems and patient injuries of the medical devices concerned.

The US FDA CGMP had been the only regulatory quality system requirements specifically for medical device until the publication of ISO 13485:1996. The European norm version of ISO 13485:1996 (i.e., EN 46001) was adopted as part of the conformity assessment prescribed in European Community' (European Economic Community was renamed as European Community in 1993), Active Implantable Medical Device Directive (90/385/EEC), Medical Device Directive (93/42/EC), and later In Vitro Diagnostic Medical Device Directive (98/79/EC) for higher-risk medical devices. US FDA replaced 1987 CGMP with Quality System Regulation (QSR) to harmonize with ISO 13485:1996 in 1997.

Following the approaches taken by the United States and the European Union, many competent authorities have decided to incorporate quality management system implementation as one of mandatory requirements for high-risk medical devices. This is analogous to the International Conference on Harmonisation (ICH) approaches. The ICH developed a harmonized guideline on pharmaceutical quality system for the lifecycle of the product and emphasized an integrated approach to quality risk management. ICH published Q9 Quality Risk Management in November 2005 and Q10 Pharmaceutical Quality System in June 2008.

The international standard, ISO 13485:2003, medical devices—quality management systems—requirements for regulatory purposes, as the title suggested specifies quality management requirements for the medical device sector for regulatory purposes. By the end of 2012, at least 22,237 ISO 13485:2003 certificates had been issued in 97 countries and economies. The 2012 total represents an increase of 2,388 (+12%) over 2011. The top three countries for the total of certificates were Germany, the USA, and Italy and the top three in growth since the 2011 survey were Italy, Germany, and the USA [1].

## 2. Medical Device GMP Regulation and Corresponding Measures

### 2.1. Medical Device GMP Regulation

Taiwanis one of the pioneers in Asia in its medical device regulation, which dates back to the 1970s. The Department of Health of Taiwan (DOH, later reorganized as the Ministry of Health and Welfare in 2010) revised medical device regulations in 1998 for the establishment of a risk-based regulatory system. The system classified medical devices into three classes according to their risk, promulgating mandatory medical device good manufacturing practice (GMP) regulation for medical devices, and introduction of the premarket review and postmarket controls. The revision of the regulations completed in 2005 aims to provide the government and industry with an international harmonized regulatory ecosystem to improve the safety, effectiveness, and quality of medical device marketed in Taiwan.

The DOH established the Medical Device GMP Promotion Task Force and convened the first meeting on 19 November 1996 to draft GMP regulation and supporting measures. After a series of stakeholder meetings and public workshops held in the following 18 months, DOH released medical device GMP regulation on 10 February 1999. To harmonize with the international standards at that time, DOH GMP regulation was based on ISO 13485:1996 with additional regulatory requirements on issues, such as outsourcing, adverse event reporting, and product recall. Following the establishment of medical device GMP, DOH published the regulation on “Classification and Management of Medical Devices” on 21 June 2000. More than 1,600 types of medical devices were classified into three classes according to risk level of the products. Class I medical device includes 700 lowest risk products such as tong tongue depressors, elastic bandages, and manual wheelchairs. GMP regulation is not mandatory for Class I medical device manufacturers unless their products are labeled as sterile or with measuring function. Over half of the medical devices on the Taiwan market were classified as Class II. Examples of Class II medical devices include blood pressure monitors, diagnostic X-ray system, and glucose monitoring systems. Class III medical devices are the highest risk level and subject to the most stringent controls. Typical Class III medical devices include HIV diagnostic reagents, pacemakers, and coronary stents. Manufacturers of Class II and Class III medical devices need to establish quality systems in accordance with GMP regulation before their product licenses are issued by DOH.  Table 1 illustrates the number of medical devices under each classification and GMP requirement for different classes of the products.

Medical device manufacturers were required to complete the GMP registration process by the end of a 5-year transition period (2001–2005).

The current GMP regulation was revised in 2013 by TFDA to harmonize with ISO 13485:2003.

### 2.2. Supporting Measures to Support SMEs

To assist medical device manufacturers, in particular small and medium-sized enterprises (SMEs), in understanding GMP regulation and establishing quality system within the company, DOH supported many organizations such as industry/trade associations and not-for-profit research organizations to hold training workshops and to provide consultancy services during 2000–2005.

Besides DOH, Industry Development Bureau of Ministry of Economic Affairs and Science Industrial Park Administration also provided financial and technical support to firms to establish quality system and recruit qualified employees. It was estimated that more than a dozen public training workshops supported by government departments were held and more than 500 employees were trained each year. These supporting measures were aimed at reducing the burden and resources needed for SME to comply with GMP regulation.

The majority of medical device manufacturers in Taiwan produced Class I and some Class II medical devices at the time when GMP was first enforced. It was estimated that 75% of SMEs seek outside consultation services for the establishment of quality systems. It was reported that a typical consultation for medical device GMP took 8.8 months and cost 10~15 thousand USD in average [5].

The supporting measures provided by the government, industry associations, and nonprofit organizations are still available for SMEs that would like to enter into the medical device sector.

## 3. Impact on Local SMEs Medical Device Manufacturers Brought by GMP Regulation Requirement

Small and medium-sized enterprises (SMEs) are defined by OECD as “non-subsidiary, independent firms which employ fewer than a given number of employees.” However, the number of employees of SMEs is defined differently across economies. For example, a firm employing fewer than 250 workers is designated as an SME in the European Union, while the United States considers SMEs as firms with fewer than 500 employees, and companies with fewer than 50 employees are small firms, and companies with employees below 10 are microfirms [8].

Globally, medical device industry is innovative and a technology-based sector consists of predominately SMEs. More than 80% of medical device companies have fewer than 50 employees, and many (notably innovative startup companies) have little or no sales revenue [9]. In Europe, 95% of medical device firms are SMEs [10] with the majority of the medical device companies being small and microsized, employing less than 50 people [11].

In Taiwan, SME is defined as enterprise with fewer than 200 employees. A firm which employs fewer than 5 workers is defined as small enterprise [12]. According to a government statistics in 2001, essentially all medical device manufacturers were SMEs [13].

The tightening of medical device regulation generally discourages the creation of new medical device companies and startup firms [14]. Although medical device regulation may provide quality system exemptions, they are, however, generally based upon the risk level of medical device and not on the size of company. Potential adverse impact of new regulations hence can be harmful to SMEs in particular [15]. SMEs tend to have less human and financial resources than large firms to implement the regulation and deal with additional bureaucratic processes [16]. Therefore, as would be expected, new medical device regulation tends to be more burdensome to SMEs than large firms [17]. A 2001 study in the USA showed that small business bears a disproportionately large share of federal regulatory burden [18].

The two main components of the compliance costs to the companies are as follows:

1. the time cost of internal staff on collecting, maintaining and understanding regulatory requirements, establishing procedures, completing forms and preparing the necessary information, and dealing with the relevant government authority;
2. the financial costs of external professional [19].

During the scheduling of medical device GMP regulation, oppositions and concerns were raised by industries. By analogy with the experience of pharmaceutical GMP implementation which reduced the number of local SME drug manufacturers, some policy analysts argued that quality management system regulation might raise the burden of SME medical device manufacturers and jeopardize the survival of such companies. This reduction in number of local manufacturers could lower the accessibility of lower-price medical devices, raise healthcare cost, and weaken sector's competiveness in general [20–23].

The dissentient voices might not be unfound. According to a 1961 government statistics of Taiwan, before the enforcement of the pharmaceutical factory standard in 1959, there were 468 pharmaceutical factories (not including 359 Chinese herbal medicine factories) in Taiwan. Some pharmaceutical factories failed to comply with the regulatory requirement and were not allowed to operate as a result. It was estimated that 250 pharmaceutical factories were closed down between 1961 and 1968. The number of pharmaceutical factories increased again in 1970s. It was estimated that there were 900 pharmaceutical factories in Taiwan in 1981. DOH mandated pharmaceutical GMP in 1982 and the number of factories decreased again. There were only 231 GMP registered pharmaceutical factories in 1995 [24]. Nearly two-thirds of Taiwan pharmaceutical companies discontinued operation after the enforcement of pharmaceutical GMP. Although the quality and competiveness of Taiwan pharmaceutical industry improved, the number of companies did not reach the 1980s level again [25].

### 3.1. The Development of Taiwan Medical Device SMEs after GMP Implementation

Before DOH revised medical device regulations in 2000, the differentiation between medical devices and other medical products was not clear. This might lead to underestimation of the actual scale of production and number of products in medical device industry before 2000.

There were about 200 medical device manufacturers in Taiwan in 2000, with most of them located in northern part of the island such as Taipei, Taoyuan and Hsinchu (see Table 2). Taiwan medical device industry started from making wound care products such as gauze and bandage provided by textile factories after the Second World War. Medical masks, surgical gowns, patient bed sheets, and examination gloves were the leading manufacturing products in 1960s. Metallic surgical instruments, electrocardiography (ECG), mercury sphygmomanometers, intravenous infusion sets, syringes, sutures, blood sampling devices, and scalp needles caught on in 1970s. Blood pressure monitors, hearing aids, wheelchairs, clinical thermometers, surgical operation tables, bone implants, electrical surgical knives, and transcutaneous electrical nerve stimulation (TENS) became dominated products in 2000 [26].

**Figure 1:**
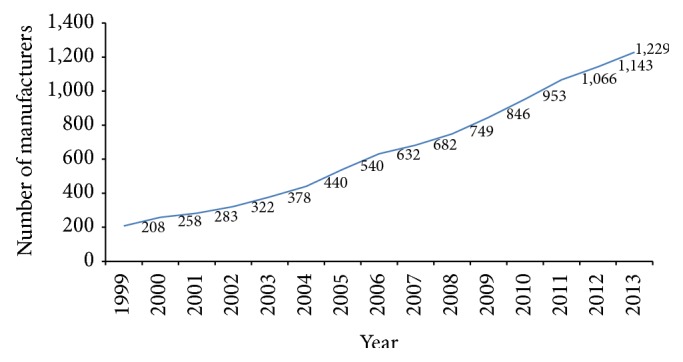
Taiwan medical device manufacturers during 1999~2013. Source [2].

According to DOH (and TFDA), there were 208 medical device manufacturers across the island in 1999. The number of registered medical device manufacturers firms increased constantly to the level of 1,229 in 2013 [2] (see Figure 1).

**Figure 2:**
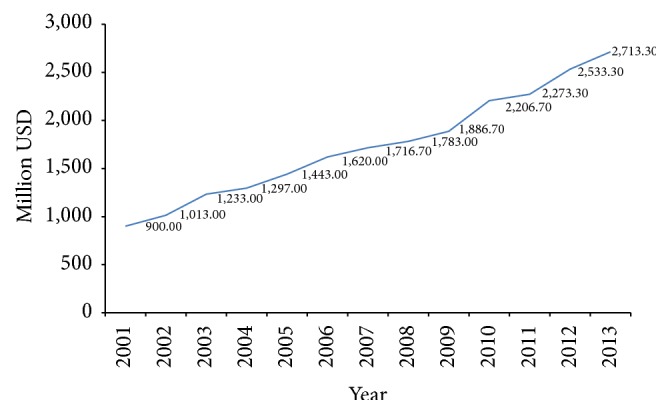
Taiwan medical device production during 2001~2013. Source [4, 6].

**Figure 3:**
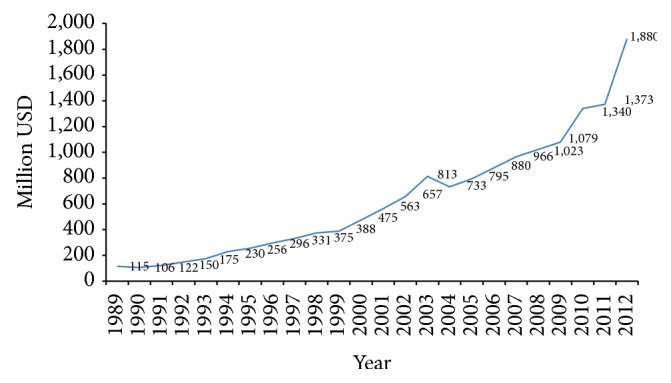
Taiwan medical device export during 1989~2012. Source [4, 6, 7].

The production and export of Taiwan medical device increased significantly in the past ten years (see Figures 2 and 3). 

### 3.2. The Development of Medical Device Manufacturer Association

With the improvement in product quality, Taiwan's medical device is more competitive globally as the growth rate of export showed, which also explained the overall growth of production of Taiwan medical device industry.

By analyzing the transition of the medical device manufacturer association in Taiwan after the implementation of GMP regulation, it helps us to understand how quality of medical device manufactured in Taiwan was enhanced as a result of the implementation of the GMP regulation.

The impact of GMP regulation on local manufacturers may also be observed from analyzing the evolution of the medical device association in Taiwan.

The current medical device manufacturer association in Taiwan was originally the “Taiwan Absorbent Cotton Industry Association” established in 1953. It was reorganized to become “Taiwan Medical Device Industry Association” (TMDIA) in 1995 and later renamed as “Taiwan Medical and Biotech Industry Association” (TMBIA) in 2013 to reflect the composition of its members [3].

In the 1970s, most of medical device manufacturers in Taiwan were family-owned company. In addition to gauze and bandages, various products such as hospital beds, wheelchairs, surgical tables, surgical lamps, dental chairs, sterilization devices, and laboratory instruments were later added into the product list of medical devices made by association members. Now, Taiwan is a major supplier of blood glucose monitoring system, powered wheelchairs/scooters, and blood pressure monitors in the world (see Table 3).

The rapid growth of Taiwan medical device industry began in 1990s. TMDIA played an important role in promoting medical device GMP. TMDIA was entrusted by DOH to assist firms in complying with regulation by providing qualified trainers and consultants to firms. Before the end of the transition period of GMP in 2005, more companies producing medical devices joined TMDIA. The membership grew by as many as 50% to 260 members in 2005. Many information and communication technologies companies developing medical devices also become members of the TMBIA in recent years. Investment in in vitro diagnostic medical devices, medical imaging system, artificial joints, dental implants, medical apps, and mobile health devices has increased significantly since 2010. Currently, there are 350 companies registered under TMBIA [3].

### 3.3. Medical Device GMP Registered Manufacturers

**Figure 4:**
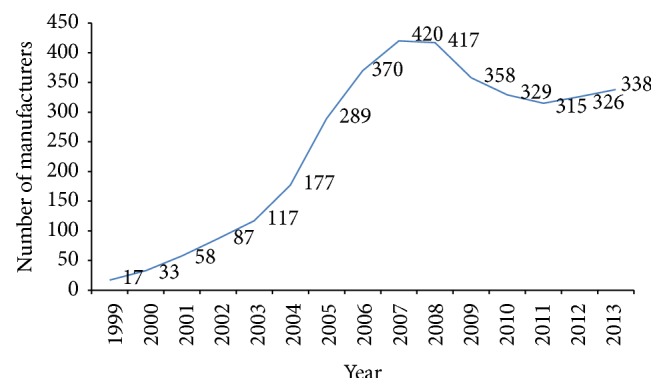
Number of medical device GMP manufacturers during 1999~2013. Source: the authors [2].

Medical device GMP requirement is mandatory for the manufacturers of Class I sterile or with measuring function, Class II, and Class III devices. In addition, Class I medical devices manufacturers may also apply for GMP registration voluntarily. The number of medical device GMP registered manufacturers was only 17 in the first year (1999) and increased to 289 at the end of the transition period in 2005, and, by 2013, the number increases to as many as 338 [2] (see Figure 4).

### 3.4. The Taiwan Medical Device Manufacturing Workforce

**Figure 5:**
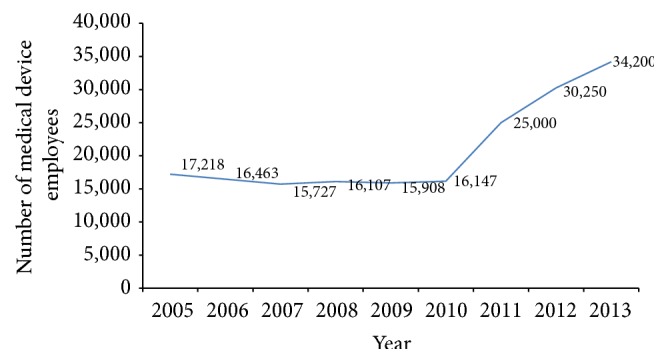
Taiwan medical device industry employees. Source [4].

In addition to the growth in number of medical device manufacturers after the enforcement of GMP regulation, the size of firms was also enlarged. The number of the workforce employed by the medical device manufacturers increased constantly from 2005 to 2013 (see Figure 5). Statistical data regarding the number of employees of medical device firms audited by Center for Measurement Standards/Industrial Technology Research Institute (one of four TFDA medical device GMP designated auditing organizations) in 2006 showed that 26% of the firms employed fewer than 10 workers, 67% of the firms hired less than 50 employees, and only 4 firms had more than 100 employees. In 2009, 25% of the firms hired less than 10 employees, 86% of the firms employed no more than 50 workers, and 3 firms had more than 100 employees. A similar data was also collected for 2013, showing that 17% of the firms hired fewer than 10 employees, 61% of the firms did not hire more than 50 employees, and 13 firms had more than 100 employees.

Some manufacturers enjoy a constant growth in business after GMP regulation enforcement. According to Taiwan Stock Exchange Corporation, examples of local large medical device manufacturers which employ more than 500 persons include ApexBio (592 employees), Kang Na Hsiung (KNH, 569 employees), Bionime (826 employees), Pacific Hospital Supply (515 employees), Microlife (3,400 employees), Taidoc (850 employees), St. Shine (2,000 employees), Rossmax (1,554 employees), and Avita (520 employees) [4].

## 4. Conclusion

The success of Taiwan medical device industry in the last decade is contributed by different factors such as global market growth, technology innovation, increased financial investment, educated human resources, and governmental initiatives.

Based on statistical data collected by the government and market survey organizations, the present paper reports the growth of Taiwan medical device industry after the enforcement of GMP regulation. As shown by the growth rates of the production and export, the international competiveness of Taiwan medical device industry is enhanced. Quality of Taiwan medical devices is recognized by global healthcare providers and consumers. Transition in the production, technologies, and number of employees of Taiwan medical device industry between 1998 and 2013 provides the competent authorities around the world with an empirical foundation for further policy development.

Policy makers around the world need to understand and evaluate the impact of regulatory requirements on the local small-to-medium-sized manufacturers and implement a harmonized regulatory system with care to mitigate the impact, thereby ensuring the success of the implementation of their policy.

The present study suggested that the implementation of GMP regulation facilitates the transformation of local medical device industry. The standard set forth by GMP regulation did not jeopardize the development of local industry as some analyst believed. Supporting measures assisting SMEs in quality system establishment and human resource improvement is essential for the success. SMEs which comply with GMP regulation can strengthen their competiveness in both domestic and global markets.

Further studies should be conducted in order to understand the key factors of GMP regulation implementation. Nonetheless, this study agrees that improving the information availability to SMEs, a friendly environment for SMEs to conduct new product development, and broad channels for SMEs to access regulatory information will ensure the success of new regulation as well as industry development [16, 27, 28].

## Figures and Tables

**Table 1:** Taiwan GMP requirement for medical devices.

Classification	Class I		Class II	Class III
Number of medical device classification names (2014)	737		936	153

GMP	Not required	Required for sterile devices or devices with measuring function	Required for all Class II medical devices	Required for all Class III medical devices

**Table 2:** Number of medical device manufacturers in 2005 and 2013.

City/county	2005	2013
Taipei City	37	56

Taipei County	197	361

Yilan County	10	17

Taoyuan County	52	145

Hsinchu City	11	26

Hsinchu County	25	58

Miaoli County	9	22

Taichung City	22	175^*^
Taichung County	45	

Changhua County	34	99

Chiayi City	2	5

Nantou County	6	13

Yunlin County	6	9

Chiayi County	10	25

Tainan City	13	109^*^
Tainan County	29	

Kaohsiung City	8	88^*^
Kaohsiung County	14	

Pingtung County	6	10

Hualien County	2	1

Keelung	2	10

Total	**540**	**1,229**

Source [2].

^*^Taichung City/County, Tainan City/County, and Kaohsiung City/County were combined in 2010.

**Table 3:** Taiwan top export medical devices during 1997~2012 (in amount of sales).

Year	Top export medical devices
1997	Examination gloves, physical therapy devices, manual wheelchairs, powered wheelchairs/scooters, hearing aids, and sphygmomanometers

2000	Powered wheelchairs/scooters, manual wheelchairs, glucose meters, artificial limbs, physical therapy devices, blood pressure monitors, medical instruments, prosthesis devices, and syringes

2002	Powered wheelchair/scooter, examination gloves, physical therapy devices, surgical devices, prosthesis devices, blood pressure monitors, manual wheelchairs, glucose meters, and contact lenses

2003	Powered wheelchairs/scooters, physical therapy devices, examination gloves, surgical devices, blood pressure monitors, prosthesis devices, manual wheelchairs, and glucose meters

2004	Powered wheelchairs/scooters, physical therapy devices, examination gloves, surgical devices, prosthesis devices, manual wheelchairs, laboratory devices, contact lenses, and blood pressure monitors

2005	Powered wheelchairs/scooters, physical therapy devices, examination gloves, surgical devices, prosthesis devices, contact lenses, glucose meters, laboratory devices

2006	Powered wheelchairs/scooters, surgical devices, physical therapy devices, contact lenses, laboratory devices, examination gloves, surgical devices, and glucose meters

2009	Powered wheelchairs/scooters, laboratory devices, glucose test strips, surgical devices, contact lenses, glucose meters, and physical therapy devices

2010	Powered wheelchairs/scooters, glucose test strips, laboratory devices, surgical devices, contact lenses, glucose meters, and physical therapy devices

2011	Glucose test strips, laboratory devices, powered wheelchairs/scooters, contact lenses, surgical devices, glucose meters, and IVDs

2012	Glucose test strips, contact lenses, laboratory devices, surgical devices, powered wheelchairs/scooters, glucose meters, and IVDs

Source [3, 4].

## Abbreviations

CGMP: Current good manufacturing practice. “CGMP requirements for devices in part 820 (21 CFR part 820) were first authorized by Section 520(f) of the Federal Food, Drug, and Cosmetic Act (the act). Under Section 520(f) of the act, FDA issued a final rule in the Federal Register of July 21, 1978 (43 FR 31 508), prescribing CGMP requirements for medical devices. This regulation became effective on December 18, 1978, and was codified under part 820.” See http://www.fda.gov/medicaldevices/deviceregulationandguidance/postmarketrequirements/qualitysystemsregulations/
DOH: Department of Health of Taiwan
GMP: Good manufacturing practice
ICH: International Conference on Harmonisation
QSR: Quality System Regulation. In 1996, FDA revised the CGMP regulation to add the design controls authorized by the Safe Medical Devices Act. The part 820 revision was published on October 7, 1996 (61 FR 52602), and went into effect June 1, 1997. See http://www.fda.gov/medicaldevices/deviceregulationandguidance/postmarketrequirements/qualitysystemsregulations/
SME: Small and medium-sized enterprise
TFDA/MOHW: Food and Drug Administration/Ministry of Health and Welfare of Taiwan
TMBIA: Taiwan Medical and Biotech Industry Association
TMDIA: Taiwan Medical Device Industry Association
US FDA: The United States Food and Drug Administration
21 CFR 820: Part 820, CFR, Code of Federal Regulations Title 21.

## References

[1] ISO (2012). *The ISO Survey of Management Standard Certifications*.

[2] Food and Drug Administration (2013). *Statistics of Pharmaceutical Affairs*.

[3] Taiwan Medical Device and Biotech Industry Association Website http://www.tmbia.org.tw/eng/.

[4] Industrial Technology Research Institute (2003–2013). *Medical Device Industry Year Book*.

[5] Huang S. *The Benefits and Difficulties of Implementing GMP for Medical Device Manufacturers in Taiwan*.

[6] http://tie.tier.org.tw/.

[7] Invest in Taiwan

[8] OECD Website (2008). *Glossary of Statistical Terms*.

[10] EUCOMED (2013). *Financial Impact of the Revision of the EU Medical Devices Directives on European SMEs and Industry*.

[12] Ministry of Economic Affairs Taiwan

[13] Department of Statistics and Ministry of Economic Affairs (2001). *List of Factories in Taiwan*.

[14] EUCOMED Factsheet: financial impact of the revision of the EU medical device directives on european SMEs and industry, 2013.

[15] Small Business Policy Branch (2003). *Small Business and Regulatory Burden*.

[16] OECD SMEs: employment, innovation and growth.

[17] Organisation for Economic Co-operation and Development (1997). *Small Business, Job Creation and Growth: Facts, Obstacles and Best Practices*.

[18] Crains W. M., Hopkins T. D. (2001). The impact of regulatory costs on small firms.

[19] Bickerdyke I., Lattimore R. (1997). Reducing the regulatory burden: does firm size matter. *Industry Commission Staff Research Paper*.

[20] Jayakumar P. B. (2008). *Small-Scale Pharma Firms Struggling for Survival*.

[21] Paul B. (2013). *Pharma Industry Demands Immediate Govt. Intervention for Survival*.

[22] Lin Y. (2005). *Milestone of Taiwan Herb Medicine—Chinese Medicine GMP*.

[23] Kuangjen C.

[24] Lin S. (2008). *Pharmaceutical Standards and Development of Pharmaceutical Trading*.

[25] Tseng L., Chang Y., Chiang C., Chen K., Chiu W., Kuo C. (2002). Taiwan pharmaceutical industry status quo and strategy. *Journal of Far East University*.

[26] Industrial Technology Research Institute *Medical Device Industry Analysis*.

[27] (2010). *United Nations Conference on Trade and Development, Integrating Developing Countries’ SMEs into Global Value Chains*.

[28] Gorlova O., Grzybowska-Brzezińska M., Żuchowski I. (2012). ISO standards and quality costs as instruments of companies’ competitive advantage. *Socio-Economic Research Bulletin*.

